# Thyroglossal Duct Cyst, a Case Report and Literature Review

**DOI:** 10.3390/diseases10010007

**Published:** 2022-01-25

**Authors:** Anas Taha, Bassey Enodien, Daniel M. Frey, Stephanie Taha-Mehlitz

**Affiliations:** 1 Department of Surgery, Wetzikon Hospital, 8620 Wetzikon, Switzerland; bassey.enodien@gzo.ch (B.E.); daniel.frey@gzo.ch (D.M.F.); 2 Clarunis, University Center for Gastrointestinal and Liver Diseases, 4002 Basel, Switzerland; Stephanie.taha@clarunis.ch

**Keywords:** thyroglossal duct cyst, review

## Abstract

A thyroglossal duct cyst (TGDC) is one of the most commonly encountered congenital anomalies of the neck. However, it is difficult to diagnose as differentiating it from other cysts like brachial cysts, lymphangiomas, epidermoid cysts, dermoid cysts, and hydatid cysts, is challenging. In this paper, we systematically reviewed the literature of 47 patients—25 males (53.1%) and 21 females (44.7%)—about their TGDC to assess the clinical picture, therapy, and prognosis of the disease. Most of the patients were children under the age of ten (63.8%). All patients had a history of a painless swelling in the anterior midline of the neck that moved in response to deglutition and tongue protrusion, thus interfering with their daily activity. Post-resection recurrence was unusual, with only 3 of 47 patients (6.4%) experiencing recurrence.

## 1. Introduction

A thyroglossal duct cyst (TGDC) is the most frequent embryonic-origin cervical mass discovered in the anterior of the neck. The thyroglossal duct, which connects the base of the tongue to the thyroid gland, generally fails to obliterate, resulting in this disease. This anomaly occurs in approximately 7% of people [1,2,3], representing about 75% of the congenital masses of the neck. Though this medical condition usually occurs in children, it is frequently discovered in young adults as well (usually in their twenties) [1,2,4]. A TGDC occasionally presents as a mobile, non-tender, non-lobular neck swelling, usually below the hyoid bone. There are some complications associated with this condition. First, an upper respiratory tract infection can quickly spread to the TGDC since they are filled with lymphoid. Additionally, it can present as a painful, large mass when accompanied by local inflammation. Second, when the cyst enlarges to the point where it bursts open, it is referred to as a thyroglossal fistula. Third, in some individuals, some thyroid gland remnants remain in the thyroglossal duct, causing a thyroid tumor to grow in a cyst, and may sometimes form a thyroid duct cyst within the thyroid gland—i.e., an “intrathyroidal thyroglossal duct cyst”—which is difficult to differentiate from a solitary thyroid nodule. It is also challenging to diagnose when this manifests on the mouth’s floor or in the sublingual area since it is difficult to differentiate it from other neck swellings like cystic hygromas, dermoid cysts, and ranula. Therefore, imaging is essential to determine a thyroglossal cyst, substantiate the diagnosis, and affirm that there is functioning thyroid tissue (if present) [5].

## 2. Materials and Methods

**Figure 1:**
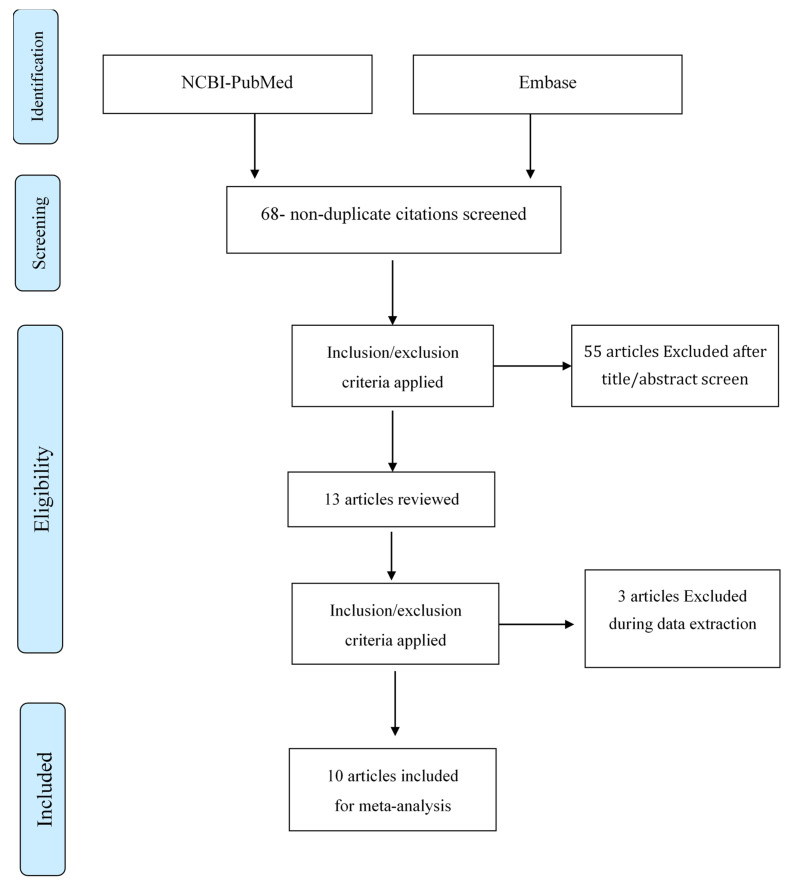
“PRISMA Diagram” depicting the flow of information across the various phases of a Systematic Review.

In this study, we conducted a systematic review of all case presentations focused on a TGDC from 1 January 2017 to the time of this study. All studies were found using the search terms “thyroglossal duct cyst case report” on Embase (https://www.embase.com) (accessed on 24 December 2021) and NCBI-PubMed (https://pubmed.ncbi.nlm.nih.gov/) (accessed on 24 December 2021). The search results showed 68 results (non-duplicated), but 58 studies were excluded after looking at the research title/abstract because they did not focus primarily on a TGDC. We included all ten remaining publications in this analysis and thoroughly reviewed and analyzed them (see Figure 1).

A total of 46 patients (excluding our patient) were studied with regard to the mass characteristics, treatment, and clinical outcome based on the 10 preselected studies [6,7,8,9,10,11,12,13,14,15]. Including our case, the study sample, therefore, included 25 males (53.1%) and 21 females (44.7%), along with one case of unknown gender (2.1%). The mean age of the patients was 29.1 years (ranging from 5 days to 85 years). Infrahyoid was the most common location for this mass with 33 cases occurring there (70.2%), followed by suprahyoid with 12 cases (25.5%), and then a further two intralingual cases (4.3%). The mean mass size was 54.4 mm (see Table 1).

## 3. Case Presentation

A 52-year-old male presented with a history of large, elastic, and easily mobile midline neck swelling and difficulty swallowing. There was no hoarseness or shortness of breath. The patient led an active lifestyle. The thyroid gland was normal in size but showed certain signs of inflammation. The patient was advised to reduce his stress and treat the swelling with progesterone gel. But after a while, he complained that those measures were not working as the mass size was fixed and did not change in size. The patient also complained of not being able to button up his shirt (which indicates the large size of the mass).

The patient had regular bowel movements, a normal appetite, slight weight loss, and no heart disease. He suffered from hypertension but was not on medication. He experienced some bloating and cramps in the abdomen, without allergy. The patient was a non-smoker but was an occasional drinker. His family history showed that his mother suffered from hyperthyroidism and his father suffered from arterial hypertension.

On physical examination, a large (0.041 m × 0.044 m × 0.033 m), regular, non-tender, and easily mobile suprahyoid cystic mass was found in the upper neck area, located above the larynx, which moved with swallowing. The thyroid gland was easy to palpate.

**Figure 2:**
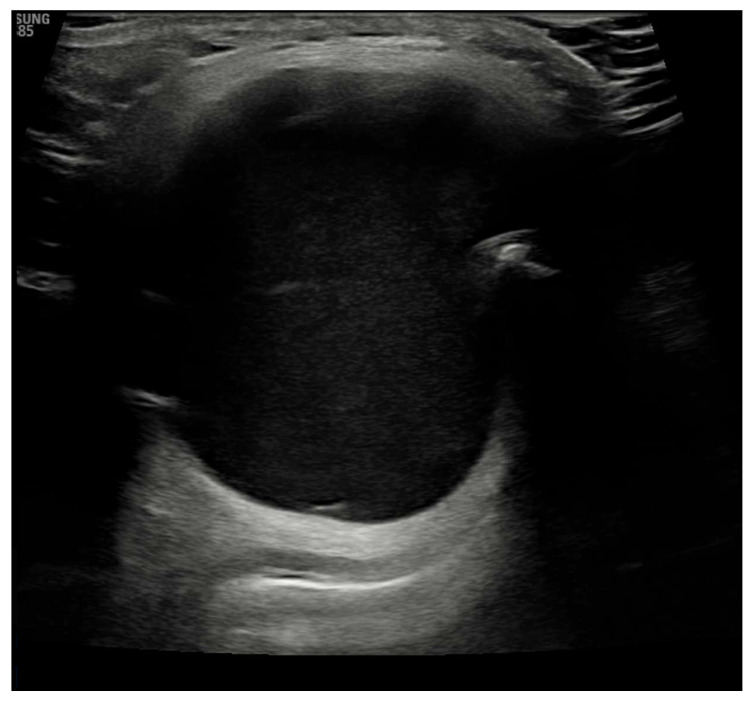
Ultrasound image showing echo-normal and homogeneous thyroid gland.

Laboratory results showed that the patient was euthyroid, and his anti-thyroid peroxidase (anti-TPO) was negative. Ultrasound imaging showed an echogenic, homogeneous thyroid with a total volume of 7 mL, with no intra-thyroid nodules. It also showed a mass above the thyroid gland, slightly to the right of the midline. The mass was approximately 0.041 m × 0.044 m × 0.033 m with a strong connective tissue capsule and an anechoic internal structure, as shown in Figure 2.

**Figure 3:**
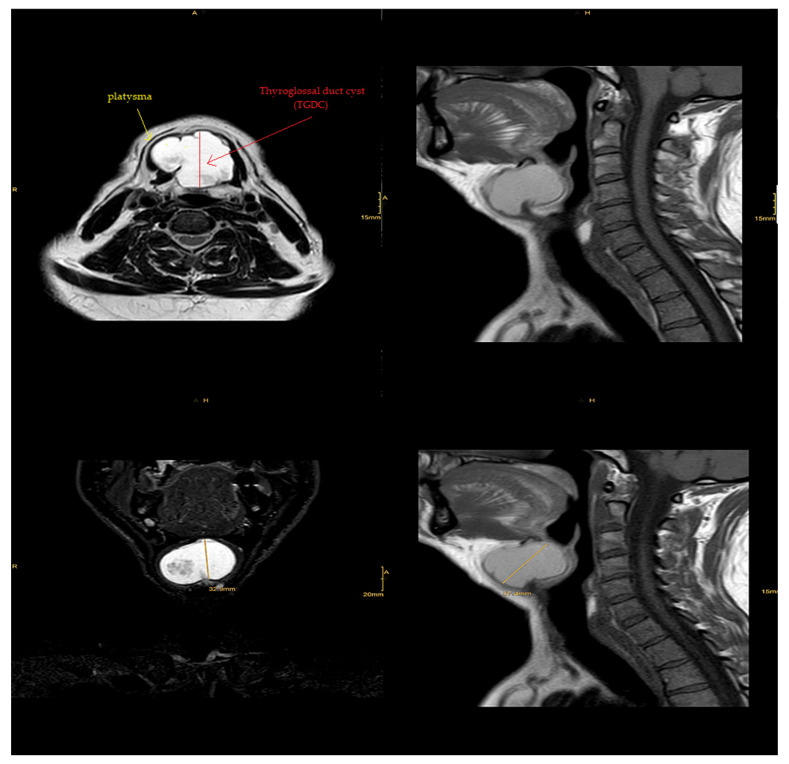
MRI image of the neck’s soft tissues (native and IV KM) showing suprahyoid thyroglossal cyst located posterior to the platysma.

Magnetic resonance imaging (MRI) of the neck’s soft tissues (native and IV KM) diagnosed it as a suprahyoid thyroglossal cyst located posterior to the platysma, as it appeared hyperintense in T2 with isointense parts and was also isointense in T1 with an extension of approximately 0.041 m × 0.044 m × 0.033 m. There was minimal displacement of the hypopharynx and epiglottis with no evident connection to the thyroid gland or the tongue’s base. There was also discreet contrast-medium uptake of the wall and protein-rich fluid, indicating possible previous inflammation. We found no cervical lymphadenopathy, and the submandibular gland and parotid gland were inconspicuous. In the cervical spine, as shown in Figure 3, no significant degenerative changes could be identified.

## 4. Results

According to the literature review of the study, a total of 47 patients were reviewed, with 25 males (53.1%), 21 females (44.7%), and one instance of undetermined gender (2.1%). They ranged in age from 5 days to 85 years old (mean 29.1 years). The majority of the patients were under the age of 10 (63.8%). The clinical behavior of the TGDC was typically benign, with recurrence after resection uncommon (only 3 of 47 patients (6.4%) recurred throughout the documented follow-up). The mass size at diagnosis averaged 54.4 mm (range, 26–120 mm). The majority of the patients had a common case history of painless swelling in the anterior midline of the neck that moved in response to deglutition and tongue protrusion and interfered with their daily activity. Infrahyoid was the most common tumor site, accounting for 33 cases (70.2%), followed by suprahyoid (12 cases, 25.5%), and two intralingual cases (4.3%).

Typically, a TGDC is a mass in the neck that is flexible and located on the midline or a bit to the side (95% err slightly to the left), most often with other symptoms (that are worrisome). Some who are affected by a TGDC suffer from pain in the neck, relapse, or experience dysphagia infections in the throat [16]. The mass generally moves, thus consuming 75% of “cysts” that are situated under the bone (hyoid). We calculate the average TGDC to be sized from 2 to 5 cm based on the sizes reported [17]. Except for five cases where there were no ultrasound records, all ultrasounds showed normal thyroid glands. Before coming to the hospital, 7 of 47 patients (14.9%) had been experiencing infections and were given antibiotics for 7 to 11 days before surgery. The mass excisions of all patients were effective.

## 5. Discussion

TGDCs occur in varying locations; they can be located anywhere in the neck between the foramen cecum, the tongue’s base, and the suprasternal fissure. Four common locations are thyrohyoid (60.9%), suprahyoid (24.1%), supra-sternal (12.9%), and intra-lingual (2.1%) [18]. TGDCs are usually associated with functional impairments like dyspnea, dysphonia, and dysphagia [19]. The main symptom is an anterior neck swelling that moves with deglutition and tongue protrusion, a clinical sign that differentiates it from thyroid swelling, which moves with deglutition only. Yet, having said that, the TGDC swelling sometimes appears in a non-classical form.

In our patient, there were no functional impairments, just a neck swelling with some mechanical impairments.

A TGDC is usually a painless, mobile swelling in the neck’s midline near the hyoid bone. It also can manifest clinically or radiologically, however, as an unusual lesion, which may make diagnosis a difficult and challenging task [20,21].

There is a risk of infection with or without an abscess when thyroglossal fragments remain attached to the tongue’s base via the tract, which can represent the start of a thyroglossal cyst’s presentation [22]. In our patient, MRI showed signs of previous inflammation. As that could have been the cause of the cyst enlargement, we leaned toward a diagnosis of a thyroglossal cyst.

The abscess of the thyroglossal cyst may drain into the neck’s skin either after an improper incision ending in a fistulous tract, or drain through the sinus tract to the tongue’s base, thus creating a solution containing fibrous residue. In the end, for the true cyst to remain or develop, a healthy capsule must form, but the infected duct remains chronically [23].

There are several methods for diagnosing a TGDC, including ultrasound imaging of the neck, an appropriate and non-invasive procedure. Preoperative fine-needle cytology is also a cheap and safe method [24]. Ultrasound imaging of our patient showed an echogenic, homogeneous thyroid with a total volume of 7 mL, with no intra-thyroid nodules. It also showed a mass measuring 0.041 m × 0.044 m × 0.033 m above the thyroid gland with a solid connective tissue capsule and an anechoic internal structure.

However, some of the disadvantages of ultrasound imaging (sonography) lie in the fact that it does not visualize hyoid and infrahyoid TGDCs reliably, and cannot reliably measure the base of the tongue in a suprahyoid cyst. MRI is often preferred for an athyroid cyst near the tongue’s base [24]. On T2-weighted MRI images, a typical TGDC) appears as a huge cyst with characteristic upward tapering and a hyperintense tract spreading to the tongue’s base. That was somewhat close to our patient’s MRI, which supported the preoperative diagnosis of a TGDC.

On reviewing the literature, the presence of a mass in the neck was the only evidence based on which to diagnose the condition during the examination of the case. With laboratory investigation, the final diagnosis indicated a suprahyoid thyroglossal cyst located posterior to the platysma, with an extension of approximately 0.041 m × 0.044 m × 0.033 m.

## 6. Conclusions

Even in adult patients, it is important to be aware of congenital anomalies that might lead people to develop neck masses such as thyroglossal duct cysts (TGDCs), and their complications, so that proper investigations can be performed to reach a definitive diagnosis. After reviewing the patient’s condition, the data revealed the presence of a mobile mass in the neck area, with no further clinical data other than swallowing difficulties. Thus, we performed a thyroid function test to make sure the thyroid was working properly, which showed that the gland was unaffected. Ultrasound imaging later revealed a mass above the thyroid gland, ruling out intra-thyroid nodules, but the mass remained unclear. Finally, we employed magnetic resonance imaging (MRI), which showed similar results to the typical TGDC, and thus the final diagnosis was made based on MRI data.

## Figures and Tables

**Table 1:** Demographic data of the study population.

Factors		Frequency
Age (years)	≤10	30 (63.8%)
	11–20	9 (19.1%)
	21–30	2 (4.3%)
	31–40	1 (2.1%)
	41–50	2 (4.3%)
	51–60	2 (4.3%)
	81–90	1 (2.1%)
Sex	Male	25 (53.1%)
	Female	21 (44.7%)
	Unknown	1 (2.1%)
Site of the cyst	Infrahyoid	33 (70.2%)
	Suprahyoid	12 (25.5%)
	Intralingual	2 (4.3%)

## Data Availability

The datasets used and/or analyzed in the current study are available from the corresponding author on reasonable request.
